# Histological evaluation of cardiac remodelling in equine athletes

**DOI:** 10.1038/s41598-024-67621-6

**Published:** 2024-07-19

**Authors:** L. C. Nath, A. Saljic, R. Buhl, A. Elliott, A. La Gerche, C. Ye, H. Schmidt Royal, K. Lundgren Virklund, T. A. Agbaedeng, A. Stent, S. Franklin

**Affiliations:** 1 University of Adelaide, Adelaide, Australia; 2 University of Copenhagen, Copenhagen, Denmark; 3 St Vincents Institute Medical Research, Fitzroy, Australia; 4 University of Melbourne, Parkville, Australia

**Keywords:** Cardiac hypertrophy, Atrial fibrillation, Cardiac hypertrophy, Atrial fibrillation

## Abstract

Approximately 1–2 per 100,000 young athletes die from sudden cardiac death (SCD) and extreme exercise may be associated with myocardial scar and arrhythmias. Racehorses have a high prevalence of atrial fibrillation (AF) and SCD but the presence of myocardial scar and inflammation has not been evaluated. Cardiac tissues from the left (LAA) and right (RAA) atrial appendages, left ventricular anterior (LVAPM) and posterior (LVPPM) papillary muscles, and right side of the interventricular septum (IVS-R) were harvested from racehorses with sudden cardiac death (SCD, n = 16) or other fatal injuries (OFI, n = 17), constituting the athletic group (ATH, n = 33), and compared to sedentary horses (SED, n = 10). Horses in the ATH group had myocyte hypertrophy at all sites; increased fibrosis at all sites other than the LAA; increased fibroblast infiltration but a reduction in the overall extracellular matrix (ECM) volume in the RAA, LVAPM, and IVS-R compared to SED horses. In this horse model, athletic conditioning was associated with myocyte hypertrophy and a reduction in ECM. There was an excess of fibrocyte infiltration and focal fibrosis that was not present in non-athletic horses, raising the possibility of an exercise-induced pro-fibrotic substrate.

## Introduction

The cardiovascular adaptations to training observed in human athletes are collectively referred to as athletic heart syndrome. These changes include a decreased resting heart rate, expansion of the plasma volume and increased cardiac chamber size^1^. Athletic heart syndrome improves athletic performance through an increase in stroke volume and cardiac output, thus improving oxygen delivery to the tissues. However, emerging evidence in human athletes suggests that remodelling is not entirely benign and could parallel pathological heart disease, with features such as fibrosis increasing the risk of cardiac arrhythmias^1,2^.

Myocardial fibrosis is characterised by increased deposition and volume of myocardial collagen and serves as a structural substrate for arrhythmias^3–6^. The mechanisms leading to myocardial fibrosis are complex and incompletely understood but it is probable that a combination of wall stress and repeated stretch of myocytes contribute to myocardial inflammation and necrosis with replacement by collagen and adipose tissue (fibrofatty infiltration)^3,7^.

It is difficult to determine whether exercise training is associated with cardiac fibrosis in ostensibly healthy human athletes because it is seldom reasonable to obtain samples for direct histological assessment. Circumstantial evidence for the presence of exercise-induced fibrosis has been derived from imaging studies of fibrosis in which delayed enhancement on magnetic resonance imaging is prevalent in athletes but not in non-athletic subjects^8–11^. A second source of evidence is derived from experimental work in rodents^4,5,12^. Treadmill exercise training in rodents results in increased cardiac chamber size, myocardial fibrosis, and arrhythmia inducibility^4,5,12^. Horses are a valid large animal model for exercise-induced myocardial remodelling that could provide additional insights into the changes involved in this process^13–15^. The horse is bred and trained for athletic performance and athletic horses consistently demonstrate exercise-induced cardiac adaptation, characterised by expansion of the plasma volume^16^, increased chamber size^17^, reduced contractility at rest^17^ and decreased resting heart rate^18^. Vagally mediated arrhythmias are common in athletic horses and are thought to be associated with intrinsic cardiac electrical remodelling^18,19^. Pathological arrhythmias such as atrial fibrillation (AF)^20–22^ and complex ventricular arrhythmias^23,24^ occur frequently in young horses in the absence of clinically detectable structural heart disease. The rate of sudden cardiac death (SCD) in athletic horses is estimated to be approximately 124–158 deaths per 100,000 horse years^25,26^ which greatly exceeds the estimates of 1 in 15,000 to < 1 in 100,000 human athlete years^27–29^.

The aims of this study were to evaluate microstructural changes in atrial and ventricular tissue from athletically trained horses, including horses with SCD and those that died from other fatal injuries (OFI) and to compare these to untrained, sedentary horses. The hypothesis of the study was that athletic horses will have cardiac microstructural remodelling including fibrosis, altered extracellular matrix composition, myocardial hypertrophy, altered capillarity, and fibroblast infiltration, compared to untrained, sedentary horses. Secondary hypotheses were that these changes will be more pronounced in athletic horses with SCD compared to OFI and that in both athletic and sedentary horses, myocardial fibrosis would increase with age.

## Results

### Horses

**Figure 1:**
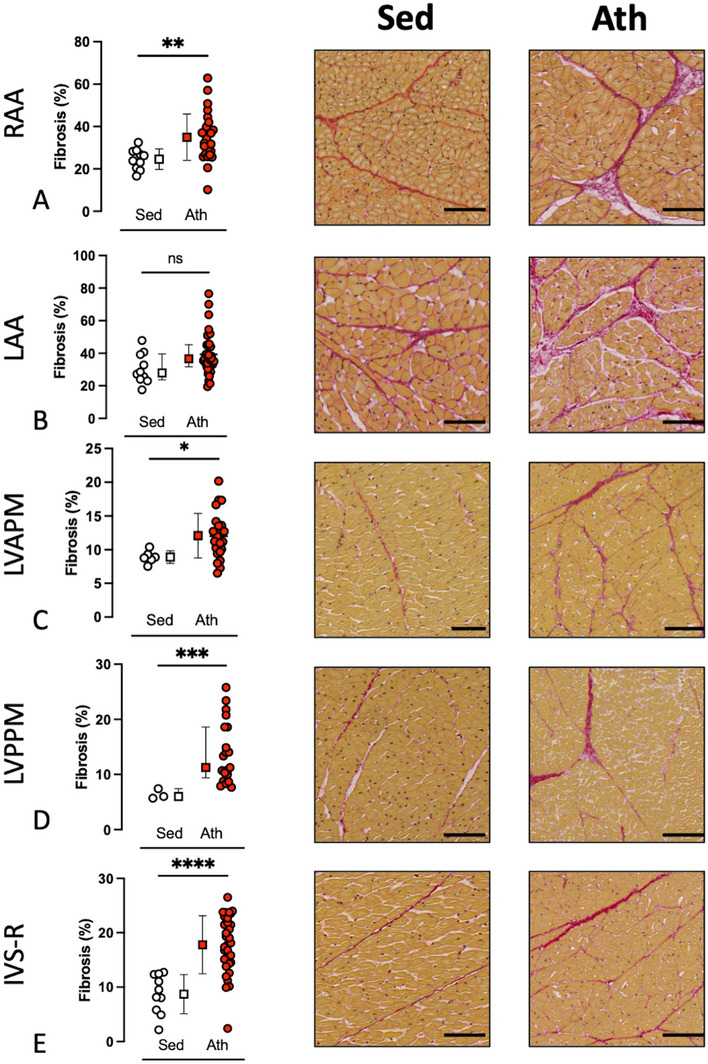
Proportion of fibrosis at each examined site for the sedentary and athletic horses. LAA = left atrial appendage, RAA = right atrial appendage, LVAPM = left ventricular anterior papillary muscle, LVPPM = left ventricular posterior papillary muscle, IVS-R = interventricular septum from the right. Charts display dotplots of the mean and standard deviation, for normally distributed data, or median and interquartile range, for non-normally distributed data, for each individual horse in each group. Normally distributed data (B, C, E) were compared using two-tailed, unpaired t-test. Non-normally distributed data (A, D) were compared using the Mann–Whitney U-test. ns P ≥ 0.05, * P < 0.05, ** P ≤ 0.01, ***P ≤ 0.001. Representative images of Sirius red stained tissues for Sedentary (Sed) and Athletic (Ath) horses are provided. Scale bar 100µm.

Sudden cardiac death horses comprised 5 females, 8 castrated males and 3 entire males, with a mean ± SD age of 4.4 ± 1.2 years (Fig. 1). Other fatal injuries horses comprised 6 females, 5 castrated males and 6 entire males, with a mean ± SD age of 4.0 ± 0.96 years. Sedentary horses comprised 5 females and 5 entire males, with a mean ± SD age of 4.8 ± 1.8 years.

### Comparison of myocardial structure between ATH and SED horses

**Figure 2:**
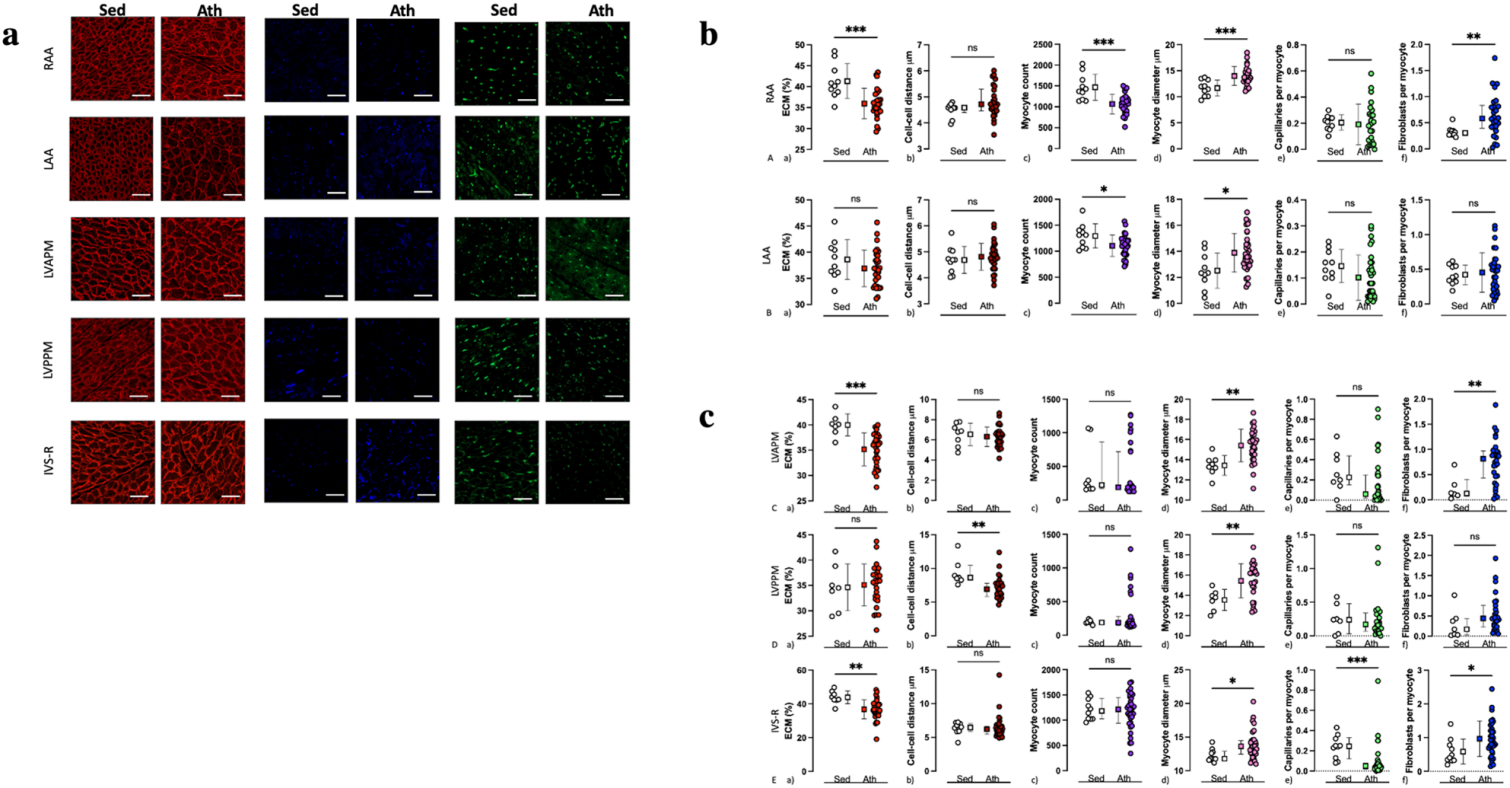
Results from WGA stained tissue. Representative images using WGA are shown in red, Vimentin shown in blue and GS-IB4 shown in green for sedentary and athletic horses. Scale bar 100 µm. LAA = left atrial appendage, RAA = right atrial appendage, LVAPM = left ventricular anterior-lateral (subauricular) papillary muscle, LVPPM = left ventricular posterior-medial (subatrial) papillary muscle, IVS-R = interventricular septum from the right, for the sedentary (Sed) and athletic (Ath) horses. (**b**,**c**) Comparison for proportion of (a) extracellular matrix (%ECM), (b) Cell to cell distance in μm, (c) myocyte count, (d) myocyte diameter in μm, (**e**) capillaries per myocyte, and (f) fibroblasts per myocyte at the two atrial sites (2b) and three ventricular sites (2c). Charts display dotplots of the mean and standard deviation, for normally distributed data, or median and interquartile range, for non-normally distributed data, for each individual horse in each group. Normally distributed data were compared using a two-tailed, unpaired t test. Non-normally distributed data (A, e; B, b, e; C, c, e, f; D, b, c, e; E, b, d, e) were compared using the Mann–Whitney test. ns P > 0.05, * P ≤ 0.05, ** P ≤ 0.01, ***P ≤ 0.001.

Sudden cardiac death and OFI groups were combined to form the ATH group and this was compared to SED horses. The results of these analyses are provided in Table 1, Figs. 1 and 2. Increased percentage of total fibrosis in ATH compared to SED horses was observed at the following sites: RAA, LVAPM, LVPPM and IVS-R. The proportion of extracellular matrix was reduced in ATH compared to SED horses at the following sites: RAA, LVAPM and IVS-R. Cell–cell distance was reduced in ATH compared to the SED group at the LVPPM. Myocyte count was reduced in ATH compared to the SED group at the LAA and RAA. Myocyte diameter was increased in ATH compared to SED horses at all examined sites. Capillaries per myocyte was reduced in ATH compared to the SED group at the IVS-R. Fibroblasts per myocyte were increased in ATH compared to the SED group at the following sties: RAA, LVAPM and IVS-R.

**Table 1:** Comparison of cardiac microstructural changes for combined group 1 and 2 (Athletic, ATH) horses and sedentary (SED) horses.

Variable Median (IQR)	N = 33 ATH horses	N = 10 SED horses	P value
Fibrosis			
LAA^#^	36.61 (31.7–45.2)	27.88 (23.7–39.6)	0.06
RAA	35.00 ± 11.0	24.58 ± 4.9	**0.007**
LVAPM	12.09 ± 3.3	8.91 ± 0.9	**0.03**
LVPPM^#^	11.25 (9.4–18.6)	6.01 (5.7–7.4)	**0.001**
IVS-R	17.79 ± 5.3	8.72 ± 3.6	** < 0.0001**
%ECM			
LAA	36.9 ± 3.5	38.6 ± 3.8	0.19
RAA	36.00 ± 3.6	41.32 ± 4.1	**0.0005**
LVAPM	35.15 ± 3.2	39.99 ± 2.2	**0.0006**
LVPPM	35.10 ± 4.1	34.61 ± 4.6	0.79
IVS-R	36.77 ± 5.7	43.86 ± 3.8	**0.002**
Cell–cell distance			
LAA	5.00 ± 0.35	4.69 ± 0.52	0.51
RAA^#^	4.72 (4.5–5.3)	4.6 (4.4–4.7)	0.14
LVAPM	6.69 ± 0.9	6.54 ± 1.1	0.56
LVPPM	6.93 (5.8–7.8)	8.63 (8.3- 5.8)	**0.003**
IVS-R	6.2 (5.5- 6.7)	6.47 (5.9–7.1)	0.48
Myocyte count			
LAA	1107 ± 207.2	1295 ± 232.6	**0.02**
RAA	1066 ± 236.6	1466 ± 310.8	**0.0002**
LVAPM^#^	189.6 (162–717.4)	223.4 (166.4–861.3)	0.51
LVPPM^#^	188.6 (147.6–275.2)	189.8 (183–220.2)	0.77
IVS-R	1171 ± 350.1	1212 ± 210.7	0.73
Myocyte diameter µm			
LAA	13.89 ± 1.5	12.52 ± 1.3	**0.01**
RAA	14.00 ± 1.8	11.65 ± 1.5	**0.001**
LVAPM	15.41 ± 1.6	13.43 ± 0.99	**0.002**
LVPPM	15.43 ± 1.7	13.54 ± 1.0	**0.009**
IVS-R^#^	13.62 (12.49–14.5)	11.79 (11.6–12.88)	**0.02**
Capillaries/myocyte			
LAA^#^	0.08 (0.03–0.2)	0.15 (0.1–0.2)	0.08
RAA^#^	0.19 (0.05–0.3)	0.21 (0.2–0.2)	0.49
LVAPM^#^	0.06 (0.02–0.3)	0.23 (0.1–0.4)	0.11
LVPPM^#^	0.17 (0.07–0.3)	0.24 (0.03–0.5)	0.71
IVS-R^#^	0.05 (0.02–0.08)	0.25 (0.1–0.3)	**0.0001**
Fibroblasts/myocyte			
LAA	0.45 ± 0.3	0.42 ± 0.1	0.73
RAA	0.64 ± 0.4	0.33 ± 0.09	**0.01**
LVAPM^#^	0.81 (0.43–0.97)	0.12 (0.07–0.40)	**0.007**
LVPPM^#^	0.44 (0.23–0.76)	0.17 (0.03–0.44)	0.07
IVS-R	0.97 ± 0.5	0.59 ± 0.4	**0.04**

According to the Shapiro–Wilk test, data were normally distributed, reported as mean ± standard deviation and compared by the unpaired t-test. Data marked by ^#^ were non-normally distributed according to the Shapiro–Wilk test, reported as median (interquartile range) and were compared by the Mann Whitney test. Significant differences < 0.05 are marked in bold.

### Comparison of myocardial structure between SCD, OFI and SED horses

A summary of the comparison between SCD, OFI and SED horses is provided in supplementary Table 1. Analysis of total fibrosis showed no differences between SCD horses and OFI horses for any of the examined variables at any of the sites.

### Relationship between myocardial fibrosis and age

**Figure 3:**
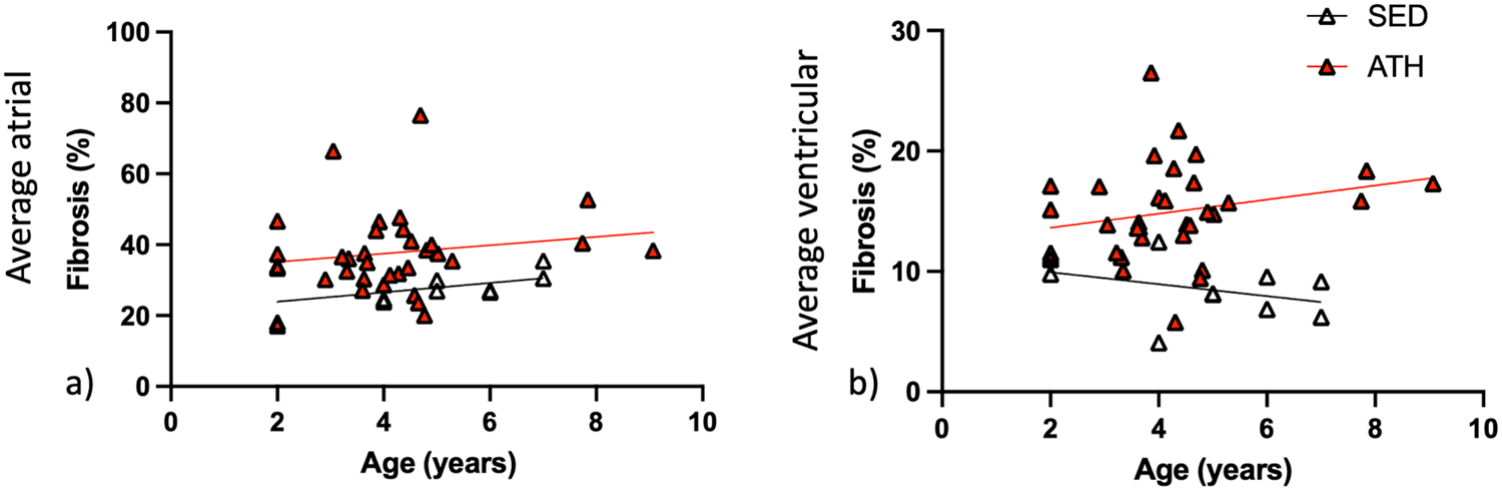
Simple linear regression analysis for the relationship between fibrosis (%) on the y axis and age on the x axis for horses in the athletic (ATH) and sedentary (SED) groups. (**a**) Average of atrial sties, left atrial appendage and right atrial appendage. (**b**) Average of ventricular sites, left ventricular anterior papillary muscle, left ventricular posterior papillary muscle and interventricular septum from the right.

Age was not found to significantly predict average atrial or ventricular myocardial fibrosis (%) for ATH or SED horses (Fig. 3). The fitted regression model for average atrial fibrosis for ATH horses was Y = 1.18*X + 32.77 (%), [R^2^ = 0.03, F (1,31) = 0.80, P = 0.38], and for SED horses was Y = 1.324*X + 21.26 (%), [R^2^ = 0.21, F (1,8) = 2.10, P = 0.19. The fitted regression model for average ventricular fibrosis for ATH horses was: Y = 0.58*X + 12.46(%), [R^2^ = 0.06, F (1,32) = 2.09, P = 0.16], and for SED horses was: Y = − 0.50*X + 10.93(%), [R^2^ = 0.14, F (1,8) = 1.28, P = 0.29], which were not statistically significant.

## Discussion

In comparing the myocardium of athletic and sedentary horses following acute death we found that athletic horses had greater myocardial hypertrophy, fibrosis and evidence of fibroblast infiltration in the majority of atrial and ventricular tissue sections. Furthermore, in some of the sections from the athletic horses, decreased ECM, cell–cell distance, myocyte count and capillary density were observed when compared with sedentary horses. This suggests that repeated exercise training may promote microstructural remodelling and myocardial scar.

Animal studies have been employed to provide potential insights into human athletic remodelling but evidence to date has largely been derived from small animals^4,5,12^. Treadmill exercise training in rodents results in increased cardiac chamber size, myocardial fibrosis and increased arrhythmia inducibility^4,5,12^. Fibrosis of the left and right atria have been observed, alongside increased inducibility of atrial fibrillation^4,5,12^. In a further rodent study, intense exercise resulted in a disproportionate increase in right ventricular mass as well as right ventricular fibrosis, changes in ventricular function and increased ventricular arrhythmia inducibility^12^. Thoroughbred horses are a large animal model for athletic cardiac conditioning and have been used to demonstrate exercise-induced cardiac enlargement but the possibility that exercise could also promote myocardial inflammation and fibrosis has not previously been evaluated^13–15^. The athletic horses in the present study were trained with routine methods^30^ including ridden exercise on a track, treadmill training and swimming. Histological analysis was performed using a standardised post-mortem protocol^31^ and a novel method for quantification of cardiac structure in myocardial tissue termed JavaCyte^32^. Using these techniques, we found more fibrosis and fibroblast infiltration in athletic horses as compared with sedentary horses, but no differences were observed between horses with SCD and horses with OFI. This might suggest that the observed fibrosis is not related to the cause of death but is associated with repeated bouts of athletic training. A smaller recent study also observed mild interstitial fibrosis or multifocal inflammation both in horses with sudden death (8 of 37) and in horses with other fatal injuries (3 of 35)^33^.

In horses, profound increases in systemic mean arterial pressure to 200 mmHg and pulmonary mean arterial pressure to 80 mmHg^34^ are generated during exercise, which provides a strong stimulus for cardiac remodelling. Strenuous exercise training induces expansion of the plasma volume by 30% compared to pre-training values^16^. In addition, the spleen of the horse stores a large volume of blood which, at the onset of exercise, can acutely augment the circulating volume by an additional 25–30%^35^. Thoroughbred racing is a predominantly aerobic event with 70–90% of energy being derived from aerobic pathways, similar to a 1500–5000 m human race^36^. Thoroughbred races are short in distance and duration, approximately 1000–3000 m and approximately 1–3 min in duration. Training volumes are highly variable and maximal intensity training is rarely undertaken more than twice per week^30^. Notwithstanding the short duration of Thoroughbred racing events and variation in training volumes, adaptive cardiac remodelling is consistently observed in horses of this discipline within 4–9 months of beginning training^17,37^. Cardiac remodelling in horses is characterised by cardiac chamber hypertrophy and is influenced by age, race distance, and race type^17,37,38^. Similar to human athletes, adaptive cardiac remodelling in athletic horses may not be entirely benign.

The pathways mediating the development of myocardial fibrosis in horses are not yet known. In humans, a limited number of imaging and biopsy studies have shown increased fibrosis in some intensively trained athletes which is suspected to reflect pathological cardiac remodelling^3^. The underlying mechanisms involved in this process are thought to include silent myocarditis, pulmonary artery pressure overload and prolonged exercise-induced repetitive micro-injury. Given the extent of gross cardiac remodelling, alongside extreme pulmonary artery pressure elevations and regular strenuous exercise participation of athletic horses, a degree of microstructural myocardial fibrosis was anticipated in this species. Myocardial fatigue after endurance races has been demonstrated in horses with echocardiography^39,40^. Elevations in cardiac troponin (cTn) following races of short duration provide further evidence of myocardial injury associated with exercise^41,42^. Mild elevations in cTn are occasionally observed at rest in athletic horses and this likely reflects chronic, low level myocardial inflammation as part of athletic conditioning^41,43^. Exercise-induced myocardial inflammation could progressively result in myocyte necrosis and replacement by collagen^3,7^. In the present study, no association was found between age, used as a surrogate to quantify the amount of exercise training, and average myocardial fibrosis in either the atria or ventricles. The potential to detect this association was limited by the small number of horses in each group. Additionally, age was estimated based on dentition in the sedentary group, which lacks accuracy compared to confirmed birthdate in the athletic horses. All horses in the present study were young and apparently healthy. The appearance of fibrosis even in young horses might indicate that myocardial fibrosis develops rapidly in response to exercise. However, further work is needed to determine the role of exercise in promoting a pro-arrhythmic myocardial substrate in horses.

Increased fibrosis in athletic compared to sedentary horses in the RAA and in all ventricular sites was identified. Fibroblast infiltration was observed in the RAA, IVS-R and the LVAPM. In rodents, whilst high intensity athletic training consistently induces myocardial fibrosis within the atria^4,5,12^, longer term training appears necessary to induce ventricular fibrosis, particularly in the right ventricle^12^. Findings of the present study suggest that fibrosis and fibroblastic infiltration occur throughout the myocardium of athletic horses, with possible sparing of the left atrium. High pulmonary arterial, pulmonary artery wedge and pulmonary capillary transmural pressures are observed in strenuously exercising horses and right and left atrial pressures also increase markedly^34,44^. Prevalence of valvular regurgitation, especially of the tricuspid valve, increases with athletic training in horses^17,37^. Compared to resting values, the proportional increase in pressure load of the right atrium and ventricle is greater than the left atrium and ventricle^45^. This proportional increase in pressure could put greater stress on the right side of the heart and might partly explain the sites most affected by fibrosis in this study.

Cardiac magnetic resonance imaging (CMR) can be used to identify myocardial fibrosis in ostensibly healthy human athletes. Patchy fibrosis in the region of the interventricular septum or left ventricular lateral wall have been reported in middle-aged endurance athletes with a prevalence ranging between 12 and 48% but is generally not observed in non-athletes^3,8,9,11^. The fibrosis in each of these CMR studies tends to be almost exclusively confined to the mid-myocardial layers, thereby suggesting a non-ischaemic mechanism. In the horse heart, it was not possible to reliably evaluate the spatial distribution of fibrosis. At a single site, the right side of the interventricular septum, a significant reduction in capillaries per myocyte was observed in the athletic horses. It could be speculated that such a reduction in capillarity might result in ischemia of the surrounding myocardium and replacement fibrosis. However, a reduction in capillarity was not significant at any of the other examined sites. Atherosclerosis, the most common cause of ischemia in humans, is not known to occur in horses. Thus, like in humans, the fibrosis in this study cannot be readily attributed to ischaemia.

Myocardial hypertrophy, as measured by myocyte diameter, was observed in athletic compared to sedentary horses at all sites, but the most pronounced difference was observed in the right atrium. The right atrium was also amongst the sites with the most pronounced elevations in fibrosis and fibroblast infiltration. This provides further support to the concept of a disproportionate increase in load on the right side of the heart during exercise and suggests that the right heart is more vulnerable to the pathological effects of exercise-induced changes in cardiac wall pressure^46^. The horse could prove to be a novel animal model for further investigation of the relative impact of high cardiovascular pressures on cardiac hypertrophy in athletes.

The present study is limited by the differences in body composition between athletic Thoroughbred horses and the sedentary wild horses. Thoroughbred horses make up a substantial portion of the genetic pool of wild Australian horses, with other breeds such as military horses and ponies also present^47^. Wild Australian horses are genetically most similar to Thoroughbred and Arabian horses^47^. Wild Australian horses differ from Thoroughbred horses in their daily activities and diet. The diet of wild horses was found to be less than optimal, with deficiencies in copper and zinc^48^. Despite dietary deficiencies, the wild horses were considered to be healthy and maintained a similar body condition score to racehorses^48^. Wild horses in Australia cover distances of approximately 18 km per day, mostly at the walk, which would not be expected to generate the high systemic and pulmonary arterial pressures that are thought to promote cardiac remodelling in racehorses^49^.

A further limitation is the difficulty in definitively classifying horses with SCD and OFI. There was potential for misclassification in that horses were included in the SCD group if fatal cardiac arrhythmia could not definitively be excluded. Thorough post mortem mitigated this risk as much as possible. Additionally, horses in the OFI group were selected based on age and sex matching and those with ambiguous causes of fatality were not included (Supplementary Fig. 1).

Myocardial hypertrophy is characterised by an increase in myocyte size^50^. In the present study, it was difficult to quantify the extent of myocardial hypertrophy in the athletic horses, given that this breed of horse is larger than the sedentary control group^51,52^ and might be expected to have comparatively larger cardiomyocytes. Additional microstructural changes indicated that myocardial hypertrophy in athletic horses occurred at the expense of surrounding extracellular stroma. A decrease in cell–cell distance was seen at a single site, the LVPPM. As has been previously performed in horses, the ECM can be used as a surrogate measure of interstitial fibrosis^15,53^. However, the ECM comprises both structural and non-structural proteins forming a complex meshwork^54^. These components include hyaluronan, hyalectans, proteoglycans, fibronectin and integrin^54^. For the present study, tissues were stained with Sirius red, to selectively highlight collagen networks and thus provide a more specific marker of fibrosis. With this technique, a decreased proportion of ECM was observed alongside increased fibrosis in equine athletes. This is consistent with results from healthy human athletes, in which CMR has demonstrated that cardiac myocyte volume increases at the expense of the extracellular volume^55^. This is in contrast to pathological diseases, such as hypertrophic cardiomyopathy, in which myocyte hypertrophy is accompanied by an increase in extracellular volume^56^. The findings in the present study suggest that in horses, strenuous exercise induces myocyte hypertrophy and a reduction in the ECM volume in a similar manner to human athletes.

In human athletes, very high volumes of intense exercise, regularly exceeding 12 METS per day, can induce pathological hypertrophy^57,58^. The sequelae of pathological hypertrophy can include cardiac arrhythmias, particularly AF^59^, with participation in endurance exercise being associated with a five-fold increased risk of AF^60^. Vigorous exercise transiently increases the risk of sudden cardiac death, but habitual exercise modulates this risk^61^. Most human athletes that have SCD have structurally normal hearts at autopsy, but in rare cases, myocardial fibrosis could increase fatality risk^3,62,63^. An accumulating body of evidence supports the role of repeated myocardial stretch, high wall stress and cardiac remodelling caused by endurance exercise in promoting a pro-arrhythmic substrate, especially of the atria^2,60,64,65^. Atrial fibrillation is the most clinically important performance-limiting arrhythmia in horses^20–22^. The present study demonstrates that atrioventricular remodelling occurs in athletic horses and that the microstructural changes include hypertrophy, fibrosis, and fibroblastic infiltration. Thoroughbred racehorses have a high incidence of AF of up to 4.9%^20^. There are early indications that myocardial sleeves in the caudal (inferior) and cranial (superior) vena cava might be at least as important as the myocardial sleeves in the pulmonary veins in triggering AF in this species^66–68^. Tissue characteristics favouring re- entry, including non-uniform myocardial fibre arrangement and the presence of fibroadipose tissue and myocardium free-islands, within myocardial sleeves of the cranial and caudal vena cava of horses have been observed^68^. The microstructural changes observed in the present study indicate that right atrial fibrosis exceeds left atrial fibrosis and this might impact on the development of arrhythmias, including AF, in Thoroughbred horses.

In conclusion, this investigation identified evidence of fibrosis, fibroblast infiltration, and hypertrophy in racehorses compared to sedentary wild horses. Myocardial fibrosis was identified in the right atrium and throughout the ventricles. A reduction in ECM indicated that cardiac hypertrophy occurred at the expense of surrounding stroma. Strenuous exercise appeared to promote fibrosis in this species and might disproportionately affect the right heart. In both human and equine athletes, an increase in some cardiac arrhythmias has been observed and it is plausible that the microstructural changes observed in this study provide an insight into the underlying mechanism.

## Methods

### Animals

This study complied with the Australian code for the care and use of animals for scientific purposes. Ethics approval was obtained from the Animal Ethics Committee of the University of Adelaide (animal ethics number S-2017-088). All methods were carried out in accordance with relevant guidelines and regulations and all methods are reported in accordance with ARRIVE guidelines .

A flowchart describing recruitment of horses to the study is provided (Supplementary Fig. 1). Cadaver study SCD horses and OFI horses were racehorses in active training sourced between 2017 and 2021. All SCD horses died within one hour, and all OFI horses died or were euthanised within 48 h, of racing or training. As per industry protocols, SCD and OFI horses had post-mortem examinations performed routinely. The SCD group comprised 16 horses in which the definitive cause of death was attributed, at post-mortem, to cardiac pathology, or in which fatal cardiac arrhythmia could not reasonably be excluded as the cause of death. The OFI group comprised 17 Thoroughbred horses that died or were euthanised due to non-cardiac catastrophic injury sustained during racing or training. Sedentary (SED) horses, comprised of 10 wild Australian brumbies, sourced from an abattoir in South Australia. All horses with SCD were retained in the study and horses with OFI were selected based on fresh tissue status at time of post-mortem and age and sex matching to SCD horses. Horses from the abattoir were selected based on pairs of males and females with age matching to the SCD horses. The age of abattoir horses was estimated based on dentition.

### Tissue collection

Following death or euthanasia at a metropolitan racetrack or training facility, cadavers from SCD and OFI horses were immediately transported and processed within 6–24 h. Heart tissue from SED horses was stored on ice and transported for processing within 12 h.

### Histology and immunohistochemistry

From each horse, tissues were harvested according to a previously described protocol^31^ from the following locations: left atrial appendage (LAA), right atrial appendage (RAA), left ventricular papillary muscle on the anterior-lateral (or subauricular) aspect of the ventricle (LVAPM), left ventricular papillary muscle on the posterior-medial (or subatrial) aspect of the ventricle (LVPPM), and the right side of the interventricular septum at the heart base (IVS-R). Tissues were fixed in formalin and paraffin embedded prior to sectioning at 5-µm thickness and affixing to slides. Fixed slides from each horse were transported to the University of Copenhagen for histological and immunohistochemical analysis. From each site, one slide was stained with Sirius red for assessment of total fibrosis and a second slide was stained with wheat germ agglutinin (WGA), vimentin and isolectin GS-IB4 isolated from Griffonia Simplicifolia according to the previously described method for evaluation of fibroblasts, capillarity and myocardial structure^13,32^.

### Staining techniques

#### Sirius red

Following deparaffinization and rehydration, the slides were stained according to manufacturer’s instructions with Weigert’s Hematoxylin (HistoLab) for black/blue nuclear staining and Sirius red (HistoLab) for red collagen staining and yellow cytoplasm staining. The slides were mounted with Pertex (HistoLab) and scanned on a AxioScanner.Z1 (Zeiss) slide scanner. For each slide, five transversely oriented subsets were created with a pixel size of 4000 × 4000 px. To ensure an unbiased fibrosis quantification, each subset was analysed by the Zen Intelessis (Zeiss), utilising a trained model to automatically segment the image subset into muscle, fibrosis and background. Fibrosis percentage was calculated as a weighted average of fibrosis divided by the total amount of tissue, excluding the background. Five repeated subsets were measured for each slide and an average of these subsets was reported and used for analysis.

#### Triple stain (WGA)

Following deparaffinization and rehydration, the slides were processed for antigen retrieval and were boiled for 15 min in a sodium citrate buffer (pH 6.00) and washed before they were further stained. First the slides were blocked in a blocking buffer containing Bovine Serum Albumin fraction V (Roche Diagnostics GmbH) and glycine (Sigma) in Phosphate Buffered Saline (PBS). The slides were incubated in primary vimentin antibody (Abcam, 1:150 in blocking buffer) overnight at 4 °C. Next day, following washing, the slides were incubated with the secondary antibody for vimentin (AlexaFluor 405 goat anti-rabbit IgG, Invitrogen, Thermo Fisher Scientific, 1:200 in blocking buffer) and the conjugated antibodies WGA (AlexaFluor 594 conjugate, Invitrogen, Thermo Fisher Scientific, 1:200 in blocking buffer) and GS-IB4 (Isolectin GS-IB4, AlexaFluor 488 conjugate, Life Technologies Corporation, Thermo Fisher Scientific, 1:200 in blocking buffer) at room temperature for two hours. Following washing the slides were mounted in Prolong Diamond Antifade Mountant (Invitrogen, Thermo Fisher Scientific) and stored at 4 °C until imaging. The slides were scanned using the AxioScanner.Z1 (Zeiss) slide scanner and five subsets from each slide were created, each of 3000 × 3000 px in size. Using the imageJ based tool (JavaCyte) the image subsets were analysed as described previously^32^ to gain information on the total extracellular matrix (ECM) content, cell–cell distance as an expression of interstitial distance, myocyte count and diameter as well as fibroblast and capillary count.

### Statistical analysis

Statistical analysis was performed using Graphpad Prism version 9^a^ (GraphPad Software, San Diego, CA, USA). Normality of the data was tested with the Shapiro–Wilk test. Normally distributed data are presented as mean (± standard deviation [SD]) and non-normally distributed data as median (interquartile range [IQR]).

Myocardial structure was evaluated through comparison of myocardial fibrosis (%), extracellular matrix (ECM [%]), cell–cell distance (µm), myocyte count, myocyte diameter (µm), capillaries per myocyte, and fibroblasts per myocyte between SCD, OFI and SED horses. For normally distributed data, differences between groups were compared using ordinary one-way analysis of variance (ANOVA) followed by Tukey’s test for multiple comparisons. Data that were non-normally distributed were compared using the Kruskal–Wallis test followed by Dunn’s multiple comparisons test.

Data from SCD and OFI were then pooled to comprise the single athletic group (ATH) and myocardial structure of the pooled group was compared to SED horses, using the same variables outlined above. For data that were normally distributed, an unpaired *t*-test was used for comparison and for data that were non-normally distributed, a Mann–Whitney test was used. Statistical significance was defined as a two-tailed p-value < 0.05.

Simple linear regression was used to test if age (years) significantly predicted myocardial fibrosis (%) for the ATH and SED groups. For this analysis, average atrial fibrosis was calculated by adding values for LAA and RAA and dividing by two. Average ventricular fibrosis was calculated by adding values for LVAPM, LVPPM and IVS-R and dividing by 3.

### Supplementary Information

Supplementary Information.

## Data Availability

Data was sourced from privately owned horses and permission has been granted to use these tissues for research. This data cannot be openly shared. Data can be obtained by request from the corresponding author.
