# Supplementary material for: Histological evaluation of cardiac remodelling in equine athletes
Source: Sci Rep. 2024 Jul 19;14:16709. doi: 10.1038/s41598-024-67621-6 (PMC11271503; doi:10.1038/s41598-024-67621-6)
Supplement: Supplementary file 1 — Supplementary Information. [file 41598_2024_67621_MOESM1_ESM.docx]

Title: Histological evaluation of cardiac remodelling in equine athletes

Authors: Nath, L.C., Saljic, A., Buhl, R., Elliott, A., La Gerche, A., Ye, C., Schmidt Royal, H., Lundgren Virklund, K., Agbaedeng, T.A., Stent, A., Franklin, S.

**Supplementary table 1:** Comparison of cardiac microstructural changes for sudden cardiac death (SCD), other fatal injuries (OFI) and sedentary (SED) horses. Normally distributed data are reported as mean ± SD and were compared by ordinary one-way ANOVA. Non-normally distributed data^#^ are reported as median (interquartile range) and were compared by the Kruskal-Wallis test.

| Variable  Median (IQR) | N = 16 SCD horses | N = 17 OFI horses | N = 10 SED horses | P value | SCD vs OFI | SCD vs SED | OFI vs SED |
| --- | --- | --- | --- | --- | --- | --- | --- |
| Fibrosis  LAA  RAA  LVAPM  LVPPM  IVS-R | 43.03 ± 14.1  38.6 ± 13.3  11.85 ± 3.5  15.68 ± 7.0  19.58 ± 3.8 | 35.72 ± 11.1  31.36 ± 6.6  12.39 ± 3.12  12.12 ± 3.2  16.21 ± 6.1 | 30.65 ± 9.4  24.58 ± 4.9  8.91 ± 0.94  6.38 ± 0.92  8.72 ± 3.6 | 0.04  0.004  0.09  0.03  <0.0001 | 0.20  0.12  0.90  0.27  0.13 | 0.04  0.003  0.14  0.03  <0.0001 | 0.54  0.20  0.08  0.22  0.001 |
| %ECM  LAA  RAA  LVAPM  LVPPM  IVS-R | 37.5 ± 4.2  36.12 ± 3.8  35.63 ± 3.2  36.38 ± 3.9  38.59 ± 5.1 | 36.32 ± 2.7  35.73 ± 3.6  34.71 ± 3.3  33.91 ± 4.0  34.84 ± 5.8 | 38.6 ± 3.8  41.32 ± 4.1  39.99 ± 2.2  34.61 ± 4.6  43.86 ± 3.8 | 0.29  0.003  0.002  0.30  0.001 | 0.63  0.96  0.69  0.28  0.10 | 0.73  0.006  0.01  0.64  0.06 | 0.26  0.006  0.002  0.93  0.001 |
| Cell-cell distance  LAA  RAA  LVAPM  LVPPM  IVS-R | 5.00 ± 0.35  4.84 ± 0.74  6.69 ± 0.9  6.9 ± 1.6  6.60 ± 1.1 | 4.62 ± 0.59  4.79 ± 0.38  5.95 ± 0.9  7.4 ± 1.8  6.43 ± 2.2 | 4.69 ± 0.52  4.51 ± 0.27  6.54 ± 1.1  9.4 ± 2.0  6.35 ± 0.89 | 0.09  0.31  0.09  0.01  0.91 | 0.10  0.97  0.09  0.78  0.95 | 0.28  0.31  0.93  0.01  0.91 | 0.95  0.47  0.33  0.04  0.99 |
| Myocyte count  LAA  RAA  LVAPM^#^    LVPPM^#^    IVS-R | 1060 ± 199.4  1069 ± 273.8  190.4 (158 – 1009)  224.8 (172.5 – 627.7)  1241 ± 392.2 | 1153 ± 210.5  1061 ± 187  179.4 (165.7 – 217)  175.3 (139.6 – 190.6)  1096 ± 292.8 | 1295 ± 232.6  1466 ± 310.8  223.4 (166.4 – 861.3)  189.8 (183 – 220.2)  1212 ± 210.7 | 0.03  0.001  0.63  0.08  0.41 | 0.43  >0.99  >0.99  0.08  0.41 | 0.02  0.002  >0.99  >0.99  0.97 | 0.23  0.003  >0.99  0.72  0.65 |
| Myocyte diameter um  LAA  RAA  LVAPM  LVPPM  IVS-R | 14.08 ±1.7  14.10 ± 2.2  15.25 ± 1.7  14.95 ± 1.9  13.24 ± 1.9 | 13.71 ± 1.3  13.84 ± 1.3  15.55 ± 1.6  15.88 ± 1.3  14.38 ± 2.2 | 12.52 ± 1.3  11.65 ± 1.5  13.43 ± 0.99  13.54 ± 1.0  12.25 ± 0.94 | 0.04  0.004  0.009  0.01  0.02 | 0.76  0.92  0.85  0.28  0.19 | 0.03  0.005  0.03  0.15  0.39 | 0.12  0.02  0.008  0.008  0.02 |
| Capillaries/ myocyte  LAA  RAA  LVAPM^#^    LVPPM  IVS-R^#^ | 0.13 ± 0.1  0.20 ± 0.1  0.06 (0.01 – 0.1)  0.20 ± 0.1  0.04 (0.02 – 0.1) | 0.07 ± 0.05  0.18 ± 0.2  0.10 (0.02 – 0.3)  0.31 ± 0.4  0.05 (0.03 – 0.09) | 0.15 ± 0.06  0.21 ± 0.06  0.23 (0.1 – 0.4)  0.26 ± 0.2  0.25 (0.1 – 0.3) | 0.04  0.93  0.21  0.65  0.001 | 0.90  0.96  >0.99  0.62  >0.99 | 0.08  0.99  0.23  0.91  0.002 | 0.06  0.93  0.72  0.92  0.006 |
| Fibroblasts/ myocyte  LAA  RAA  LVAPM  LVPPM  IVS-R | 0.49 ± 0.3  0.60 ± 0.4  0.90 ± 0.5  0.52 ± 0.4  0.85 ± 0.5 | 0.42 ± 0.3  0.69 ± 0.4  0.61 ± 0.4  0.59 ± 0.5  1.1 ± 0.5 | 0.42 ± 0.1  0.33 ± 0.09  0.23 ± 0.2  0.30 ± 0.3  0.59 ± 0.4 | 0.74  0.06  0.005  0.37  0.04 | 0.76  0.82  0.14  0.89  0.32 | 0.81  0.14  0.004  0.56  0.39 | >0.99  0.06  0.13  0.34  0.04 |


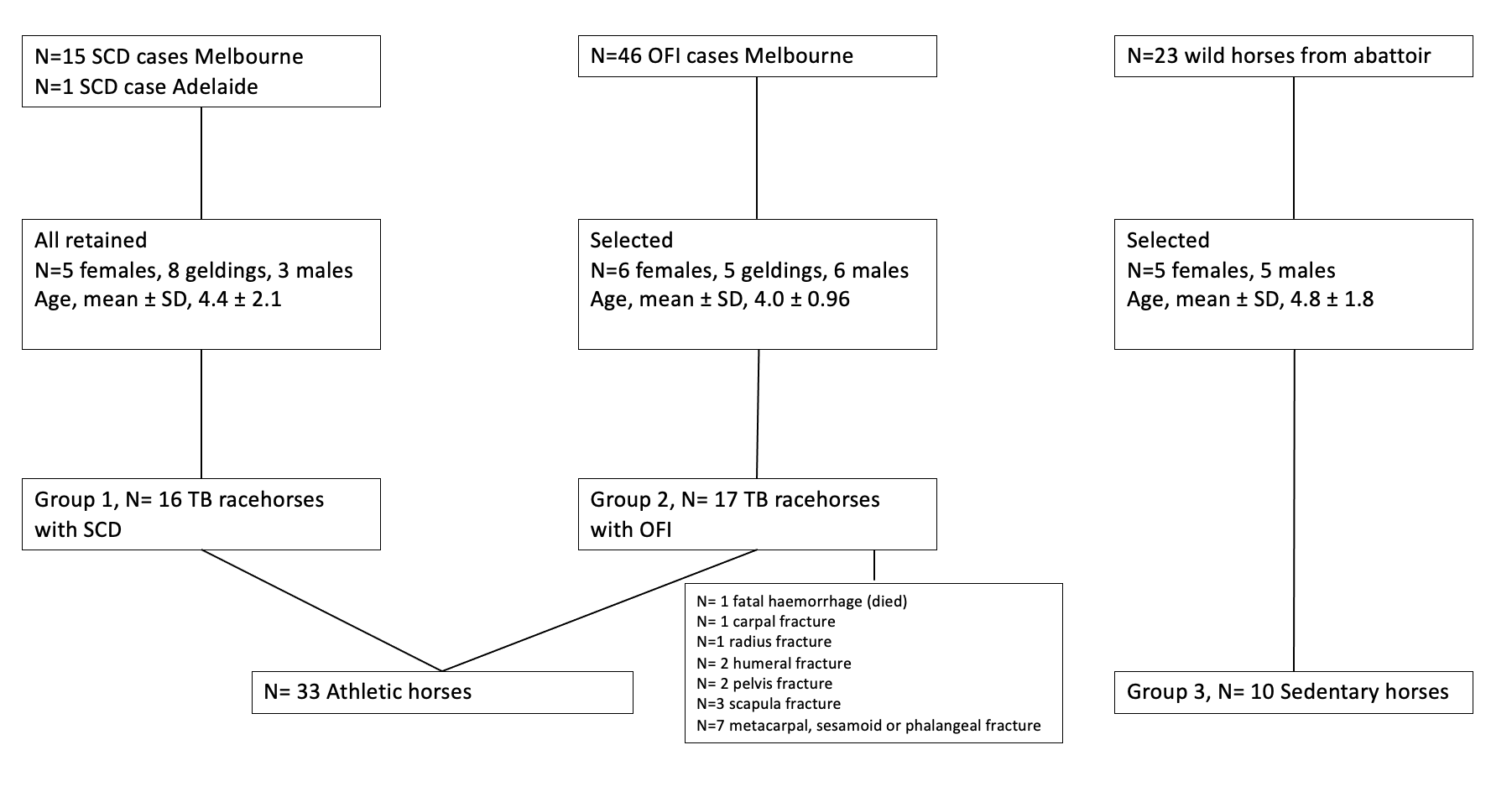


**Supplementary figure 1:** Flow chart showing the selection and classification of horses included in the study. Abbreviations: OFI = other fatal injury, SCD = sudden cardiac death, SD = standard deviation.
